# Assessing mammal trapping standards in wild boar drop-net capture

**DOI:** 10.1038/s41598-022-17407-5

**Published:** 2022-09-05

**Authors:** Carles Conejero, Jorge Ramón López-Olvera, Carlos González-Crespo, Arián Ráez-Bravo, Raquel Castillo-Contreras, Stefania Tampach, Roser Velarde, Gregorio Mentaberre

**Affiliations:** 1 Wildlife Ecology and Health Group (WE&H-https://weh.cat/) and Departament de Medicina i Cirurgia Animals, Universitat Autònoma de Barcelona (UAB), 08193 Barcelona, Spain; 2 Wildlife Ecology and Health Group (WE&H-https://weh.cat/) and Departament de Ciència Animal, Universitat de Lleida (UdL), 25198 Lleida, Spain; 3 Present Address: Fundación Artemisan, Avda. Rey Santo 8, 13001 Ciudad Real, Spain

**Keywords:** Zoology, Animal behaviour, Animal physiology, Invasive species

## Abstract

Applying contemporary trapping standards when managing wildlife should no longer be an option, but a duty. Increasing wild boar populations originate a growing number of conflicts and hunting is the only cost-effective management option in most cases. However, new scenarios where hunting is unfeasible emerge and trapping necessities cope with lacking regulatory frameworks and technical guidelines. In this research, we evaluated drop nets, a capture method not considered by the international trapping standards, to capture Eurasian wild boar (*Sus scrofa*), a wildlife species not included in the list of mammal species under the scope of the Agreement on International Humane Trapping Standards (AIHTS). Less than 20% of the captured wild boars presented moderate or severe injuries attributable to the capture method, hence fulfilling the acceptance thresholds of the outdated AIHTS. Based on the new standards thresholds of acceptance, the humaneness of drop-nets in our study ranged 66–78%, under the 85% required. The capture success and selectivity were 100%, as ensured by operator-driven triggering, which should be considered the main strengths of this method, together with the minimization of animal suffering owing the short duration of the stressful situation. Additionally, in spite of the socially adverse environment, with people contrary to wild boar removal, no disturbances against the capture system or operations occurred. This is the first assessment of a drop-net capture method according to internationally accepted mammal trapping standards, with unconclusive results. However, there is a need for adapted procedures and thresholds of acceptance aimed at not-mechanical traps in general, and specifically at drop-nets. Compared to other live-capture methods, drop-nets minimize the duration of the stressful situation —at the expense of a strong adrenergic acute response—, maximize the probabilities of capturing entire sounders of prosocial species, which may be also considered as more humane, and has the ability to coordinate higher values of capture success, absolute selectivity and adaptability to difficult environments.

## Introduction

Globally, there is a growing consensus on the need to make progresses towards ethical wildlife control, starting by considering altering the human practices that cause human–wildlife conflict and by developing a culture of coexistence^1,2^. Eurasian wild boar (*Sus scrofa* L. 1758) populations are expanding and increasing worldwide, and so does human–wild boar co-existence and conflicts. The wild boar has the broadest geographic range of all ungulates and conflicts arise due to both direct and indirect interspecific interactions resulting in ecological, economic and health impacts; namely, wild boars cause crop damages, road traffic accidents, increased risk for shared livestock diseases and zoonoses, altered food webs and damage to some plant and animal species^3–5^. Furthermore, and due to its high behavioural plasticity, the wild boar has successfully adjusted itself to a wide range of landscapes in the last decades, including urban areas^6,7^. As a consequence, wild boar population lethal control remains the only cost-effective measure to manage human–wild boar conflicts in most cases^8,9^. However, hunting is unfeasible and/or illegal in certain scenarios due to safety and/or social constraints^10^ and then other management measures must be considered^11^. The urban scene faces specific challenges in managing this species because of increasing sensitivity and social demands for animal welfare considerations^12–14^. Fertility control^15^ has been proclaimed as a more ethical alternative management measure in spite of lacking supportive evidence for its effectiveness at the population level^4,16,17^. In this respect, and according to modelling, more than 50% of the fertile females within a specific population should be sterilized to obtain meaningful reductions in wild boar numbers^18^, which is far from current technical capacities when working with abundant free-living wild boar populations^5^. At this point, live trapping and removal remains the only cost-effective and feasible option in certain scenes.

Many techniques, methods and/or devices/systems have been developed for killing and/or restraining wildlife for fur harvest, wildlife and/or related-conflicts management or with research purposes^19^. Killing traps have probably been the most used devices for both food/fur harvesting and conflict management with little, if any, consideration to animal suffering in the beginnings. Later, the increasing interest in safeguarding animal welfare led to the Agreement on International Human Trapping Standards (AIHTS), which is a binding agreement that has a direct impact on fur trading between the signatory countries^20,21^. Almost simultaneously, the International Organization for Standardization (ISO) published its own mammal trapping standards, with no legal value or enforcement capability, but voluntary^22,23^. Both the AIHTS and the ISO standards established the need for fulfilling welfare standards according to certification protocols of every intended trap previous to its authorization for mass production, commercialization and use. These certification protocols are mostly based (but not exclusively) on either time to irreversible unconsciousness, insensibility or death (TIU), in the case of killing traps, or in numerical scores that quantify the extent of injury incurred by a trapped animal in the case of restraining traps^24,25^. Mammal capture usually becomes more difficult as animal size increases, hence killing traps are mostly used to catch species ranging in size from rodents to lynx or wolf, at the most^26^, probably because of increasing difficulties to meet with welfare and performance (efficiency and selectivity) standards when used with bigger species. Although this is probably one of the raison d’être of restraining —non-lethal— traps, most certified ones according to international standards are also aimed at small to middle-sized furbearing, pest or predatory species, probably to prevent killing or damage to protected and/or endangered non-target species^27,28^. On the other hand, and for further concern, both the AIHTS and the ISO standards have been repeatedly criticized with a growing list of concerns including ineffectiveness in ensuring animal welfare due to insufficient and outdated standards and test procedures, incomplete list of mammal species included and/or lacking or insufficient thresholds of acceptance^24–26,29^. In line with this, wild boar or wild artiodactyls in general seem to be out of the scope of this regulatory framework. Very recently, new mammal trapping standards addressing the mentioned concerns have been proposed^25^. Both outdated (AIHTS and ISO) and recent standards are focused on mechanical traps —both killing and restraining— (“devices that have mechanical energy if they are in motion and/or if they are at some position relative to a zero-potential energy position” which are activated by the (hopefully) target species to be captured).

Methods developed for trapping wild artiodactyls consist mainly in restraining/live trapping systems that have been used primarily for research, population monitoring and/or translocation purposes. These include mainly different kinds of cage and corral traps, as well as net-based systems (drop-, drive- or gun-nets), also used with less attention to animal welfare in the past and increasing standards later promoted by institutional Animal Care and Use Committees, domestic regulations and/or research studies^30,31^. These methods have been mostly evaluated in terms of capture efficiency and level of case morbidity and mortality associated to the capture methods. When considered, animal welfare evaluations have been made in-vivo by means of hematological, serum biochemical and/or physiological parameters used as indicators of individual acute stress response or by attending to post-release mortality whenever possible^11,30,32–35^. More recently, the global rising trends in wild ungulate populations, especially those of wild boar in Europe^5^, are doing population control through methods other than hunting necessary. As a result, restraining methods (e.g. drop-nets) are being used as a previous step to either gunshot sacrifice or pharmacological euthanasia^11,36^. Despite animal welfare concerns are important regardless of the reason for capture, in this new context where abundant ungulate species come into the category of pest or nuisance species, the risk for double or even multiple standards exists^37^. This is even more feasible in the absence of international standards considering these species distinctive features, such as size, anatomy, behavior or use as game species, so that attention to welfare standards may differ according to operator background and expertise, as well as to requirements established for capture in every specific context. In this regard, their legal consideration as game or invasive species may further contribute to this problem due to less strict regulations compared to species with a higher level of protection.

Barcelona city is one of the big metropolises around the world suffering conflicts derived of wild boar presence in the urban structure since years ago, some of which entailing serious hazards and/or real consequences for public safety and requiring prompt measures. The Barcelona city council (BCC—*Ajuntament de Barcelona*) is involved in a multiple approach to this problem since 2013, including actions in the short, middle and long term^38,39^. Short-term actions mostly rely on live capture and removal of conflictive or potentially conflictive individuals^11^. ESTRATEKO S.L. (https://www.ESTRATEKO.com/en/) is a small enterprise founded to provide solutions in wildlife-derived conflicts management that has developed its own drop-net based system. It was the first company to be hired by the BCC to perform captures of wild boar sounders with the potential to cause conflictive situations within the urban scene. The requirements determined by the BCC include veterinarian supervision of these captures in order to minimize animal suffering and increase humaneness by applying pharmacological euthanasia instead of gunshot sacrifice (which also prevents spilled blood in capture sites where people pass-by at daytime, such as public green spaces). Properly executed, drop-net capture minimizes the duration of the stressful situation, at the expense of a strong adrenergic acute stress response^11^. According to preliminary visual inspections of both capture events and the captured wild boars, the ESTRATEKO drop-net system was considered as apparently respectful with animal welfare. Although drop-nets are not considered mechanical traps, due to the interest of ESTRATEKO S.L. in objectifying this assessment, and given the absence of specific standards and regulations, the aim of this study was to evaluate the ESTRATEKO drop-net system for wild boar capture according to available mammal trapping standards. In addition, we discuss other operational and ethical considerations.

## Methods

### ESTRATEKO remote-controlled drop-net system and operation

**Figure 1:**
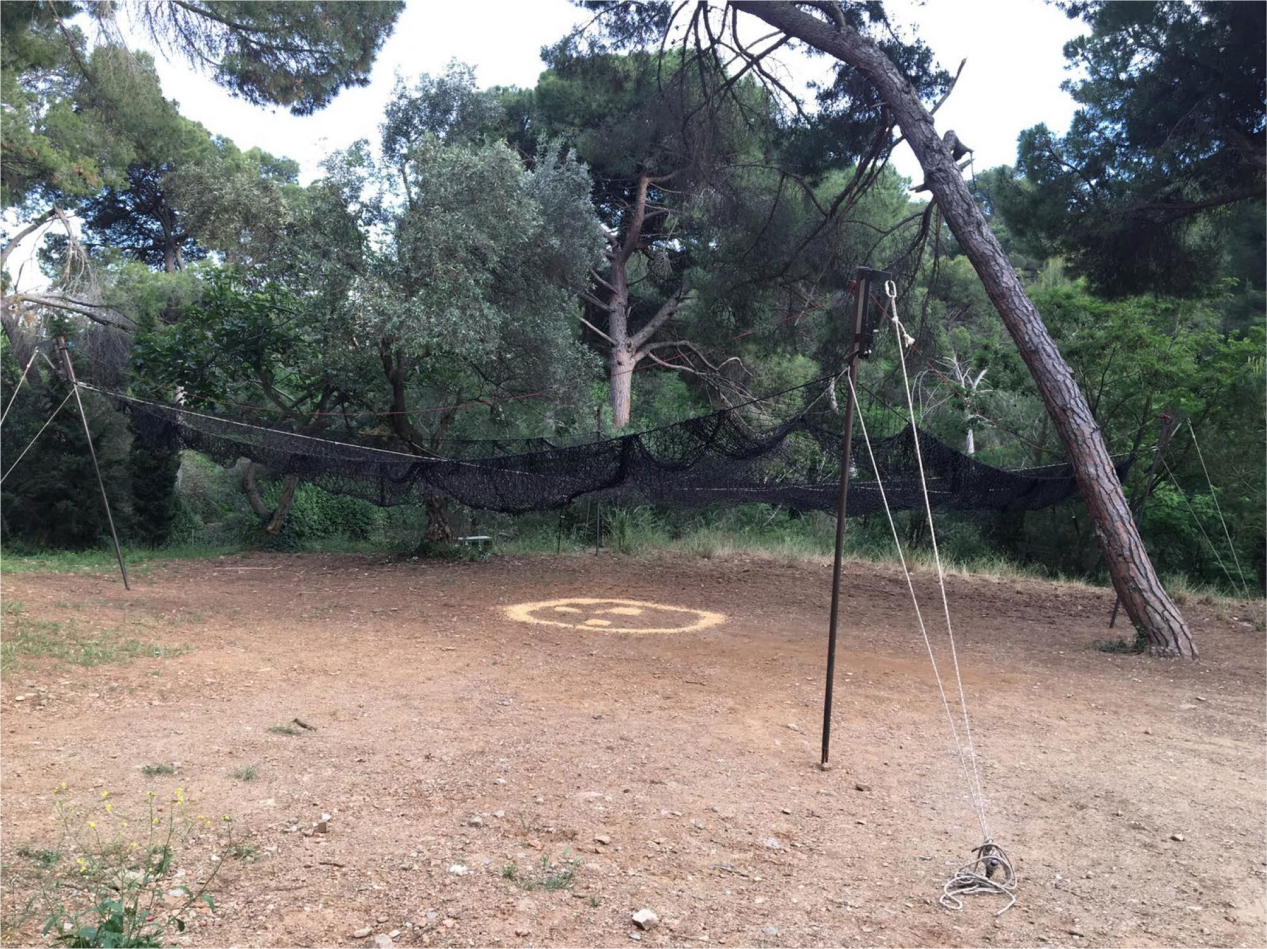
The ESTRATEKO drop-net trap deployed in a peri-urban location of Barcelona city at dusk, ready to run. Corn kernels are used as bait to attract wild boars to the central area under the net.

The ESTRATEKO drop-net system is a homemade standard drop-net device with technological improvements. Namely, the drop-net frame consists of 4 metal poles (2.5–3 m high, 10 cm in diameter), with the bottom of the poles buried 30–50 cm into the ground once placed on-site and supported by rope tension lines fastened to the top of each pole and staked to the ground (Fig. 1). The net used measures 10 × 10-m and is made of 0.5-cm diameter nylon rope displaying a 10-cm mesh made ad-hoc by a local manufacturer. The operational adaptations consist of the inclusion of an electromagnet-based system as fastening device and remote-controlled trigger mechanism, and a Wi-Fi real time video-cameras device for distance monitoring of animals’ presence under the drop net by means of a tablet and suitable software. A 12 V car battery is used for electromagnet electric supply under field conditions. Arrangement of poles allowed different and adaptable shapes and measures of the drop-net trap to the specific selected capture sites, sometimes reduced in size or with physical obstacles such as big trees.

Real-time distance monitoring is always performed on-site, in a previously selected waiting location out of sight from the trap location but less than 250–300 m away, in order both to reduce wariness around trap sites due to the operators’ proximity and to rapidly assist the captured animals after remotely triggering drop by means of the tablet software.

### Study area and capture protocol

**Figure 2:**
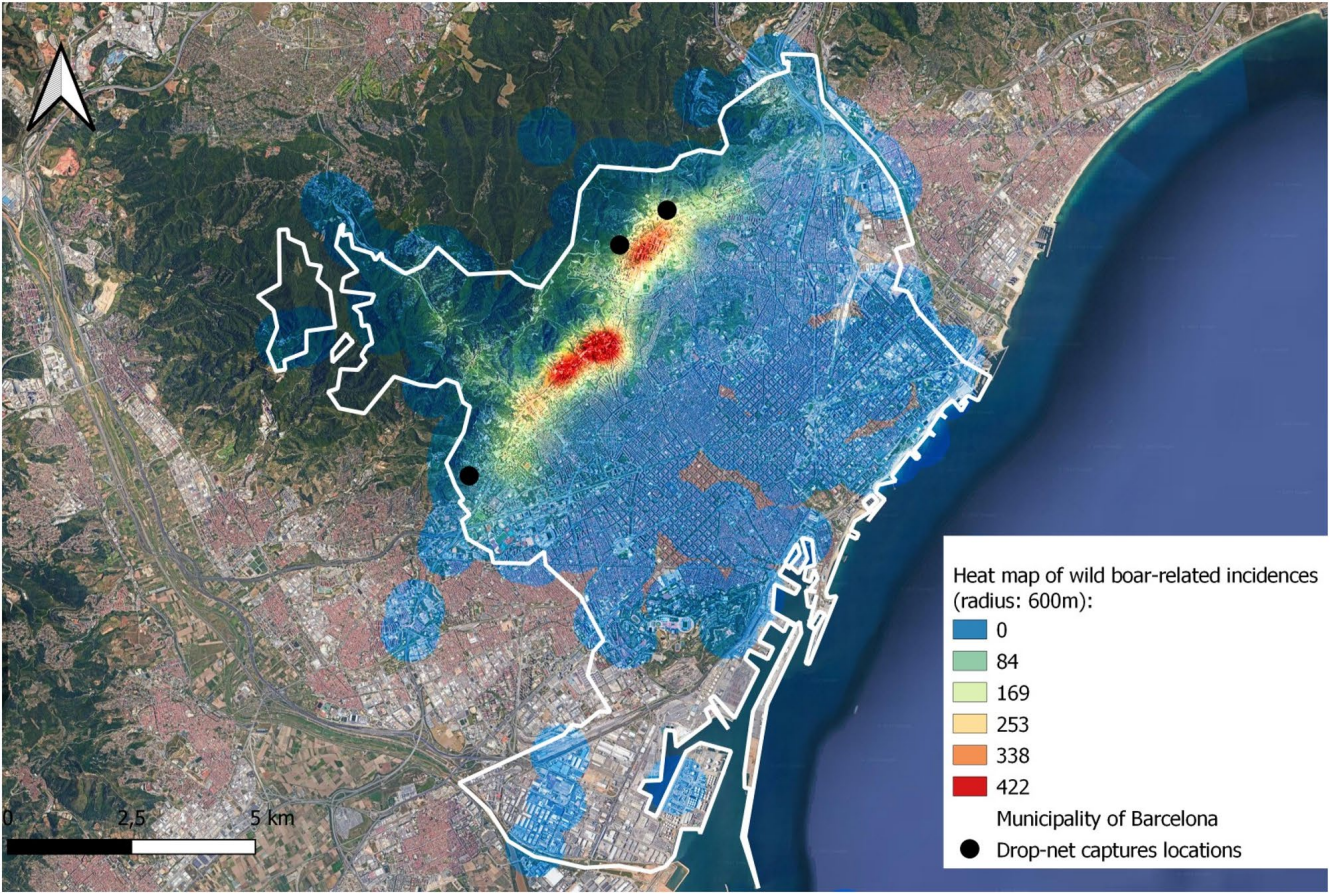
Heat map of the wild boar-related incidences recorded by the Barcelona city police department from 2010 to 2019, and approximate location of specific trap sites in the boundaries between the urban area of Barcelona and the natural area of Collserola. Generated with QGIS version 3.22.7 Bialowieza (https://qgis.org/en/site/).

From September to November 2017, the ESTRATEKO drop-net system was used in six operations in a periurban context and within the framework of the contract 16/0243-00-PR/01 with the BCC aimed at urban wild boar derived conflicts management. Three capture areas were determined at districts’ request and based on hotspots for human–wild boar conflicts in the boundaries between the urban area of Barcelona and the natural area of Collserola (Fig. 2). Specific trap sites were selected on the basis of physical characteristics (suitable for drop-nets assembly), wild boar traces and discretion (low frequented sites or with the possibility to temporary limit people access), baited with corn to promote wild boar loyalty for 1–2 weeks and monitored through infrared-triggered cameras to confirm continued daily visits by the targeted wild boars. Drop-net trap was not set during this period but only the specific days when captures were scheduled. The capture days, drop-net assembly was done before sunset, real-time distance monitoring performed as abovementioned and complete dismantling was done just after capture, the same night and before dawn.

During the capture operation, two remote cameras recorded the space beneath the suspended net and sent the live video through Internet to portable digital tablets, which allowed operators triggering the drop-net when the targeted wild boars were under the mesh, including entire sounders or family groups. Once activated, the net dropped over the targeted group or individuals, which ended up entangled and physically restrained in few seconds. Immediately, the group of operators including at least one veterinary moved to the trap site and injected the trapped wild boars in the thigh area with a mixture of tiletamine (3 mg/kg) and zolazepam (3 mg/kg) (ZOLETIL100, Virbac Animal Health, Spain) with xylazine (3 mg/kg) (XILAGESIC 20%, Calier Laboratories, Spain) prefilled in syringes according to the estimated weight of each individual. Next, wild boars were allowed to stay undisturbed and reach anesthetic unconsciousness, re-dosed when necessary, disentangled, blood sampled and finally sacrificed through intra-cardiac injection of T-61 (Merck Sharp and Dohme Animal Health, Spain). Once finished the capture operations, at late night, the animals were transported and stored in a cold room at 4 °C within the following two hours and necropsied the following day, less than 24 h after capture.

### Assessment of mammal trapping standards

Drop-nets are operator-triggered restraining traps that have not the consideration of mechanical traps^25^, hence apparently out of the scope of either currently in force mammal trapping standards (AIHTS) or the new proposed ones. However, in the absence of specific and/or alternative procedures, to assess humaneness of the ESTRATEKO drop-net system for capturing wild boars, we adopted a hybrid procedure including the pathological evaluation and trauma scoring of field —instead of compound/captive— captured wild boars, as proposed by ISO 10990-5, as well as the injury indicators and the performance threshold proposed by the AIHTS, according to national and regional regulations—also focused mainly on fur predatory species—^40,41^. Namely, we necropsied 20 (the minimum number considered by the AIHTS to obtain meaningful results) out of 32 captured wild boars. An experienced wildlife pathologist examined carcasses for the presence of injuries and/or indicators for negative effects resulting from the drop-net capture method and to evaluate animal welfare (Table 1). Next, for a more proper assessment, we discuss the results according to the recently released standards and guidelines for improvement^25^.

**Table 1:** Indicators of poor welfare to be assessed during humane trapping standards validation of capture methods, according to the Agreement of International Humane Trapping Standards (first column) and scores assigned to these lesions either by the International Organization for Standardization (second column) or by the new proposal for international mammal trapping standards (third column)^25^.

	ISO 10990-5 Annex C	New mammal trapping standards proposal^25^
**Behavioral indicators**		
Self-inflicted bite causing severe injuries (e.g. mutilation)	na	50
Excessive immobility and apathy	na	na
**Injuries**		
Fracture	30–100	50–100
Carpus, tarsus or closely related joints luxation	30	30
Tendon or ligament ruptures	25(each)–100	25(each)–100
Severe periosteum graze	30	15
Severe external or internal hemorrhage	10–100	30–100
Significant skeletal muscle degeneration	55	50
Limb (upper or lower) ischemia	55	50
Definitive tooth fracture with pulp cavity exposition	30	30
Ocular damage including corneal laceration	100	100
Spinal cord affectation	100	100
Myocardium degeneration	100	100
Amputation	25–100	30–100
Death	100	100

#### Necropsy protocol and histopathological study

For this aim, the postmortem study included the systematic necropsy of every individual, as well as a specific histopathological study. Complete external and internal evaluation was performed on all individuals in order to characterize and determine the severity of the traumatic injuries that could be related to the drop-net capture. Following external examination and before opening internal body cavities, the wild boars were completely skinned to better assess the presence of contusion injuries.

The histopathological study was mainly addressed to assess if restraining time in the net before anesthesia was long enough as to provoke capture (exertion) myopathy, which may be due to excessive stress and suffering by the captured individual. The targeted tissues for microscopical examination after fixation and staining with hematoxylin and eosin included: myocardium, skeletal muscles (*longissimus dorsi*, *semitendinosus* and/or *semimembranosus*) and kidney.

### Operational factors

A record was made of every capture event including the following data: location, date, assembly and starting time, times at which the drop-net was triggered, number and individual characteristics of the wild boars captured in every net fall, finishing time and concerning additional observations (e.g.: incidences due to unexpected opposing witnesses or another kind of disturbances detrimental to the capture event success, or operators’ safety accidents).

### Ethical statement

No ethical permit for animal experiments applies or must be permitted as no animals were harmed or killed specifically for the purposes of this study. Wild boar capture operations were done for population management purposes, not for research, and according to national and local legislation. All described methods were conducted during or after legal trapping activities, according to national, regional and local laws. All international and/or institutional guidelines for animal handling were followed. Thus, all experiments were carried out in compliance with ARRIVE guidelines.

## Results

**Figure 3:**
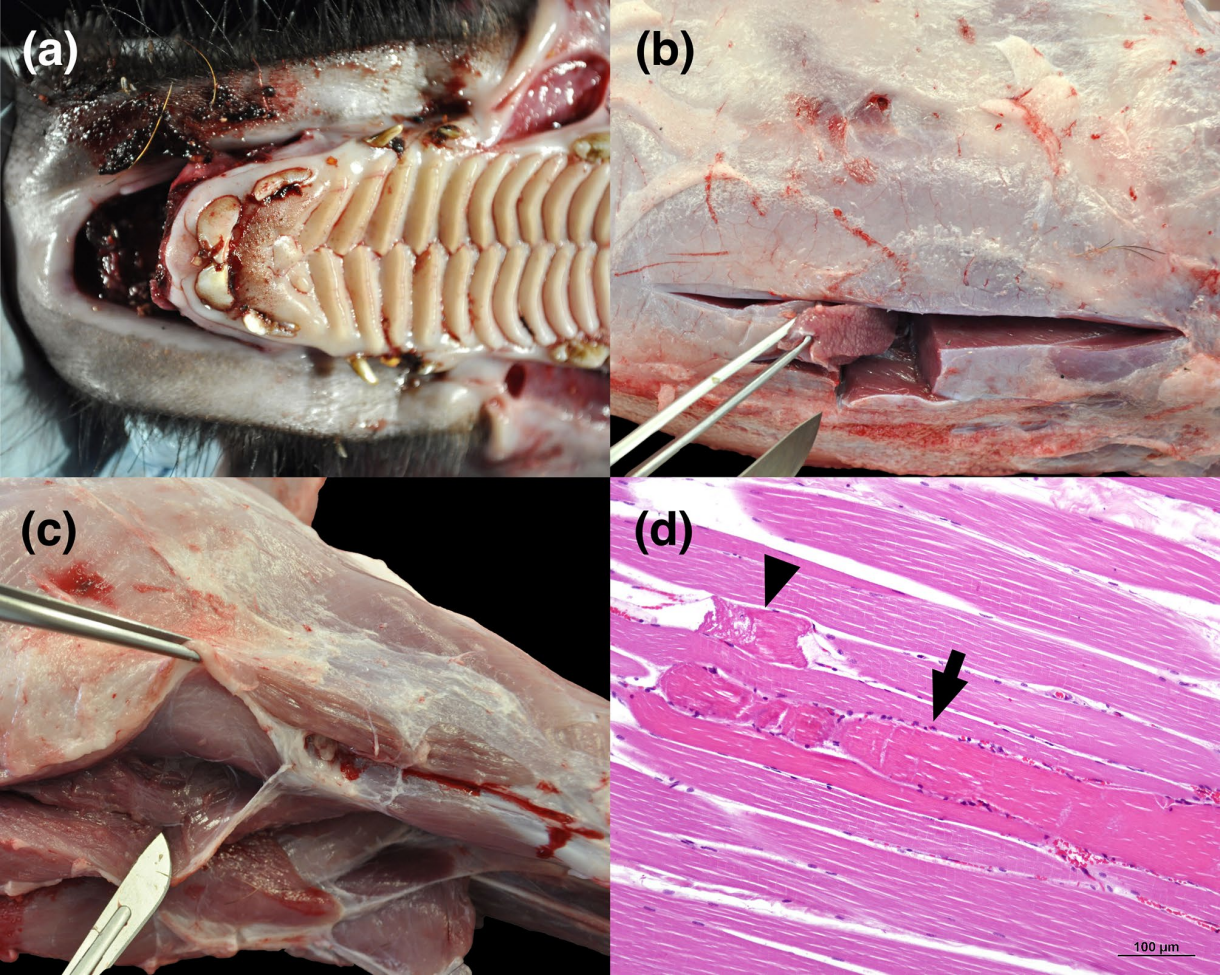
(**a**) Severe, deep gingival laceration with external haemorrhage exposing the maxillary bone. (**b**) Sampling of *longissimus dorsi*. (**c**) Sampling of *semitendinosus* and *semimembranous* muscles. (**d**) Acute skeletal muscle degeneration. A central myofiber is swollen and hypereosinophilic (arrow) and a fragmented segment of another myofiber is also present (arrowhead).

Thirty-two wild boars were captured during the six capture operations, 12 males and 20 females ranging 2 months to 6 years old and 10–90 kg. To perform the postmortem study, a partially random selection of individuals was done after every capture event to obtain representation of the six capture operations (hence, different circumstances including separate dates, locations and environmental conditions) as well as of both sexes and different age classes, until completing 20 wild boars (6 males and 14 females; same age and weight range). None of the twelve excluded wild boars displayed external signs of trauma. A male piglet suffered severe gingival laceration due to the placement and friction of the mesh nylon rope between the upper lip and the gingival space while entangling together with an adult female (its mother), which probably increased the forces between the piglet and the mesh. According to the pathological criteria established in Annex C of ISO 10990-5, this injury displayed ligament rupture, periosteum abrasion and mild to moderate external hemorrhage. In addition, two adult females caught in the same capture event displayed acute myodegenerative lesions consistent with capture myopathy. Individually, the lesions observed could be classified as mild but, under a rigorous interpretation, the simultaneous affectation of, at least, two major muscles (*longissimus dorsi* and *semitendinosus*) could be compatible with the indicator “major skeletal muscle degeneration” established by the ISO-10990-5 and classified as moderate. Hence, altogether, and depending on the interpretation, moderate to severe injuries associated with the drop-net capture were observed in one to three (5–15%) wild boars (Table 2; Fig. 3).

**Table 2:** Date, place and results from the six capture operations with the ESTRATEKO remote-controlled drop-net system. Bold characters refer to the dates, age classes, sex and number of individuals with significant trap-related injuries. *WB* Wild boar, *PM* post-mortem, A = Velòdrom d’Horta; Coordinates (UTM31N—ETRS89): 429088 E 4587916 N; B = Escola Baloo; Coordinates (UTM31N—ETRS89): 428127 E 4587226 N; C = Av. Pearson; Coordinates (UTM31N—ETRS89): 425048 E 4582627 N.

Date	Area	Captured WB	WB selected for PM study	♂Piglets	♀Piglets	♂Juveniles	♀Juveniles	♂Yearlings	♀Yearlings	♂Adults	♀Adults
				Captured/selected for PM study **(with significant injuries due to the capture system)**							
12/09/2017	A	8	4	4/1	2/1	–	–	1/1	–	–	1/1
**18/09/2017**	A	5	5	2/2 **(1)**	2/2	–	–	–	–	–	1/1
27/09/2017	A	2	2	–	–	–	–	–	2/2	–	–
**02/10/2017**	B	9	5	3/1	3/1	–	–	–	–	–	3/3 **(2)**
16/10/2017	C	4	2	–	–	1/0	1/0	–	–	1/1	1/1
28/11/2017	C	4	2	–	–	1/0	2/1	–	–	–	1/1

A fourth individual (adult female) displaying chronic gingivitis suffered moderate gingival laceration presumably due to the mesh rope friction; in this case, the previous condition was considered as a predisposing factor and was overall classified out of the ISO pathological criteria. Finally, ten individuals (including the adult female with gingivitis) displayed minor abrasions (n = 7) or contusions (n = 3) with either superficial and/or intramuscular bruise, and including an individual showing a minor bruise in the snout and another one with minor injuries in the inner side of the oral mucosa due to self-biting presumably while entangled. Minor and focal acute myodegeneration on muscular fibers associated to subcutaneous and intramuscular contusive traumatisms were observed in the histopathological study of the same individuals. Despite most of these lesions may be related to the drop-net capture system, they were all classified as mild and not to fulfil the AIHTS established severity pathological criteria. Three individuals showed previous and already healed moderate to severe traumatisms, including a partial jaw amputation, a tail amputation and a bone callus on two ribs, that were not associated to the capture method. Finally, intramyofibrillary cystic structures compatible with *Sarcocystis* spp. were observed on myocardium and/or skeletal muscle of seven individuals. See Table 3 for the detailed information of all the examined wild boars, including sex, age, condition, weight and pathological findings.

**Table 3:** Identification, mean age, weight and pathological findings on evaluated individuals. *WSL* “Without significant lesions”. *According to new mammal trapping standards proposal^25^; **According to ISO-10990-5 Annex C.

Id	Mean age (months)	Condition, weight (kg)	Trap-related pathological and histopathological findings (Injury score points*; Trauma scale**)	Pathological and histopathological findings not trap-related
SS17153	4.5	Very good, 11	WSL	WSL
SS17154	30	Good, 44	WSL	Anomaly in lower jaw (previous traumatism)
SS17155	4	Good, 15.5	WSL	WSL
SS17156	4	Good, 16	Low grade, superficial abrasion in forelimb with subcutaneous and intramuscular bruise (10; Mild)	Tail amputation (previous traumatism)
SS17162	60–72	Good, 47.5	WSL	Intramyofibrillary cystic structures on myocardium and skeletal muscle consistent with *Sarcocystis* sp. Minor perivascular lymphoplasmacytic infiltrates in the renal pelvis
SS17163	4	Good, 13.5	WSL	Minor interstitial and lymphoplasmocytic infiltrate on kidney
SS17164	4	Good, 16.5	WSL	WSL
SS17165	4	Good, 15.5	WSL	WSL
SS17166	5.5	Good, 15	Severe, deep gingival laceration (100; Severe) with external hemorrhage exposing the maxillary bone (30; Moderate) Low grade, superficial abrasion of oral mucosa (5; Mild)	WSL
SS17170	17.5	Good, 42	Self-inflicted bites on oral mucosa (5–10; Mild)	Intramyofibrillary cystic structures on myocardium and skeletal muscle consistent with *Sarcocystis* sp. Minor interstitial lymphoplasmacytic infiltrates on renal medulla
SS17171	17.5	Good, 33	Low grade, superficial longitudinal abrasion on forelimb with focal acute hemorrhagic degeneration on brachialis muscle. Erythrocytes between swollen and fragmented hypereosinophilic muscular myofibers (10; Mild)	WSL
SS17172	54	Very good, 75.5	Low grade, superficial longitudinal abrasion on forelimb with associated subcutaneous and intramuscular hemorrhage (10; Mild) Mild myodegeneration on *longissimus dorsi* and *semitendinosus* muscles with swollen and fragmented eosinophilic fibers—8%— (30; Moderate)	WSL
SS17173	4.5	Good, 11	Low grade, superficial and intramuscular bruise on forelimb with minor focal degeneration on contused muscle (10; Mild)	WSL
SS17174	54	Good, 59.5	Low grade, superficial abrasion with erythema on forelimb (10-Mild) Swollen eosinophilic and fragmented fibers—3%— on *longissimus dorsi* and *semitendinosus* (30; Moderate)	Bone callus on thoracic cavity (ribs 6 and 7) due to a previous traumatism Intramyofibrillary cystic structures on myocardium and skeletal muscle consistent with *Sarcocystis* sp.
SS17175	54	Very good, 57	Low grade contusion, superficial and intramuscular bruise in right thorax and dorsal flank with moderate focal bruise (10; Mild)	Intramyofibrillary cystic structures on skeletal muscle consistent with *Sarcocystis* sp.
SS17176	3	Good, 12.5	WSL	WSL
SS17192	54	Very good, 90	WSL	Intramyofibrillary cystic structures on myocardium and skeletal muscle consistent with *Sarcocystis* sp.
SS17193	66	Very good, 83	Low grade, superficial and longitudinal bruise on nasal bone (10-Mild)	High number of intramyofibrillary cystic structures on myocardium and skeletal muscle consistent with *Sarcocystis* sp.
SS17242	54	Very good, 59.5	Moderate grade, laceration on mandibular gingiva due to previous chronic swollen gingivitis with incisive spacing (10–30; Mild)	Multifocal coalescent pyogranulomatous glossitis, tonsilitis, lymphadenitis (submandibular and retropharingic lymphnodes), abscess (1.5 cm) caudal to mammary gland (right M3), cranioventral suppurative chronic pneumonia (10%) Intramyofibrillary cystic structures on myocardium consistent with *Sarcocystis* sp.
SS17245	7.5	Good, 18.5	Low grade, superficial abrasion on forelimb (10; Mild)	WSL

The capture success of the ESTRATEKO drop-net system was 100%; i.e. wild boars appeared and placed beneath the net in every planned capture event, so that the system was triggered and the outcome resulted in multiple captures (all the wild boars observed through the cameras were captured). The selectivity was also 100%, as ensured by operator-driven triggering. And average performance was 5.3 wild boars per capture operation (Minimum: 2; Maximum: 9; Table 2). Operator safety was warranted as no one resulted injured during the capture operations. Finally, it deserves mention that the capture system was not vandalized nor disturbances aroused during the capture operations. In no case, the trap was assembled more than seven hours (Minimum: 3 h and 30 min; Maximum: 6 h and 45 min) between 19 h and 3:15 h of the following day.

## Discussion

Worldwide, the harsh reality is that many wildlife traps continue to be widely used without any or poor evaluations of trapping standards^42^, probably due to lacking political will, regulations and supervision or control mechanisms. Even so, the need for updating the current AIHTS in order to improve animal welfare standards and test procedures has been long stated^26,29^ and recently boosted^24,25^. Some of the alleged concerns for this claim are incomplete lists of mammal species and trap types included on it. In this research, we evaluated a capture method not considered in the AIHTS to capture a wildlife species not included in the list of mammal species under the scope of the AIHTS, with apparently satisfactory results. To the best of our knowledge, this is the first assessment of welfare performance of a drop-net capture method according to international mammal trapping standards. However, as far as drop-nets are not mechanical traps, they would fall out of the scope of any standards, either the outdated AIHTS or the newly released ones^25^. Nevertheless, given the absence of specific and/or alternative standards, procedures and thresholds of acceptance, we decided to adopt the existing ones. Altogether, amongst the 20 wild boars captured and analyzed, at most three displayed significant trapping-associated alterations. This value is under the 20% maximum allowed by the AIHTS, hence the ESTRATEKO drop-net system fulfilled the requirements of this norm for restraining traps. However, the new mammal trapping standards expand on the indicators of distress by including behavioral and physiological parameters, assign scores to the injuries observed in the pathological evaluation—similarly to ISO 10990-5—and establishes stricter thresholds of acceptance. Behavior evaluation is feasible and deserves interest when live-traps do not completely restrict mobility and the captured individuals may remain for hours inside the trap or held by it. This is not the case for drop-nets, which immobilize individuals almost completely and immediate assistance is required in order to alleviate their strong acute stress response, either by gunshot or by chemical methods, depending on the intended purpose. Regarding physiological evaluation, a previous study already made clear a predominantly adrenergic stress response of wild boars captured with the ESTRATEKO drop-net system that contrasted with cortisol-induced changes observed in cage and corral trap captured wild boars^11^. Since stressful stimuli and both intensity and duration of the stress response provoked differ amongst capture methods, direct comparison and interpretation of physiological parameters may be difficult to assess unless extreme and pathological values are observed or thresholds of acceptance are determined/provided. Evaluating the results of our pathological evaluation based on the new standards thresholds of acceptance (“acceptable restraining trap systems are expected, at a 95% confidence level, to hold ≥ 85% of target animals for a specific time period without serious injuries [≤ 50 points], signs of distress or exertion [≤ 50% of the capture time], and significant physiological stress changes”^25^), only the male piglet suffering severe gingival laceration clearly scored over 50 points. The points/score to be assigned to the two individuals displaying skeletal muscle degeneration deserves further discussion. While the ISO 10990-5 differentiate minor from major skeletal muscle degeneration, scoring 30 (Moderate) and 55 (Moderately severe), respectively, the new standards proposal considers a single category of skeletal degeneration scoring 50 points. Since both individuals presented other mild affectations, their cumulative injury score with the new standards would be over 50, resulting altogether also in three unsatisfactory captures and 17 successful ones out of 20, which corresponds to a 66% humaneness efficacy (95% CI; estimated through the normal approximation to the binomial distribution, according to Proulx et al. 2020^24^ and 2022^25^), under the 85% threshold. Even doing a lax interpretation and considering only the piglet with severe gingival laceration, we would not exceed the established threshold (78%). On the other hand, the short duration of restraint during drop-net capture, as well as for other nets-based capture systems, should be considered a plus^43^ and adapted trapping standards, thresholds and evaluation procedures could be desirable.

It is worth notice that when evaluating welfare standards and performance of a capture system, it is not only the device that is under examination, but also the way it is used^26,44^, which altogether could be referred as the capture method or set. This is specially so in the case of drop-nets given that the trapped animals must be attended immediately and cannot remain unattended for hours, as it may happen with other methods to live-capture wild animals that are normally allowed to work overnight and revised next morning^42^. In this regard, we suggest that a combined test procedure of both killing and restraining traps could be advisable when using restraining traps for population control and as a previous step to sacrifice or euthanasia. Namely, the parameter “TIU” should be taken into consideration, probably with adapted thresholds to the specific live trapping method. This reasoning has been elaborated later than the experimental phase of the present study, hence we did not precisely record times from drop-net triggering to anesthesia-induced unconsciousness of the captured wild boars. However, we can assure that in no case more than 15–20 min passed between these two moments. If these data were available and we could fine-tune this assessment, we may could find a relation between the TIU values and the two wild boars displaying myodegenerative lesions compatible with capture myopathy. Going beyond, we previously stated that the wild boars captured in the present study were sacrificed by means of pharmacological euthanasia after previously induced anesthesia in order to increase humaneness of process and avoid blood spill in the periurban scene. If we omit aesthetic concerns, gunshot or captive bolt followed by exsanguination would probably have resulted in shorter TIU values in the drop-net captured wild boars, which are subjected to a highly stressful situation previous to anesthesia. Furthermore, physical immobilization provoked by drop-nets makes possible this operation in suitable conditions and safely for the operators. The two adult females observed with capture myopathy were captured in the event with a higher number of captured wild boars, which probably resulted in extended time to anesthesia. Capture myopathy is a time-dependent syndrome that normally correlates with the duration of an overwhelming (that overcome the physiological mechanisms to cope with) stressful event^34,45^. The significance of capture myopathy probably differs depending on the fate of the animals, being specially concerning if the captured animals are to be released (e.g. after marking and/or sampling); not so much if sacrificed. Hence, the operating protocol should consider increasing the number of skilled operators and/or limiting the number of wild boars to be captured before triggering the system to minimize the time of handling and prevent capture myopathy in the case of live-trapping and release^11^, whereas capture of entire sounders must be prioritized when capturing for population control. On the other hand, exceptional injuries such as that of the male piglet suffering severe gingival laceration while entangling together with a much bigger and heavier individual, despite infrequent may be avoided using a thicker and softer mesh or reducing mesh hole size. Considering the relative position of individuals beneath the net before triggering the system could serve also to prevent crowding under the net and during the entangling phase and to reduce post-triggering handling time.

Drop-nets were first conceived to capture game birds and later adapted and widely used to capture ungulates, mainly ruminant herbivores. According to the literature, drop-nets are considered suitable for mass capture of ungulates smaller than an antelope (*Antilocapra americana*) and recommended to capture deer (roe—*Capreolus capreolus*-, red—*Cervus elaphus*- and white-tailed—*Odocoileus virginianus*-deer), European mouflon (*Ovis aries*) and mountain goats (*Oreamnos americanus*) in non human-dominated scenarios^21,46,47^. However, to our knowledge, its use in free ranging swine has been quite limited, restricted to feral/wild pigs in North America^48^ and only once reported in European wild boar^11^. Other capture methods targeting wild boar such as corral or cage-traps have been proposed as one of the safest, most efficient and humane means of capturing wild pigs, also allowing for the safe release of non-target captures^14^. However, these methods have also been reported to provoke severe injuries, and even related mortality, in a significant number of individuals^30,42^. This discrepancy is probably due to differences in the design of the specific devices evaluated and in the way they are used. In fact, measures to reduce injuries and fight-or-flight responses when using these methods have been described, resulting also in quicker delivery of chemical immobilization drugs via darting and, hence, shorter time values until unconsciousness^49^. Furthermore, trap-related physical injuries may not fully reflect other capture-induced stressors such as fear, pain and poor environmental conditions^42^. Even more, the stressful situation provoked by capture has also been described to cause distress in individuals other than the ones captured in prosocial species such as wild boar^50^. Probabilities for this situation to occur are higher when using methods with less ability to capture entire sounders of prosocial species than drop-nets.

In addition to welfare considerations, a capture method must warrant capture success or trap performance (i.e.: the rate at which a device or system catches the intended species)^33^. Capture success may be estimated according to efficacy, or percentage of successful capture events, and efficiency, or number of captured individuals per unit of capture effort, which were maximum in our study, with 100% of successful capture events and 100% of multiple captures (i.e., more than a single wild boar captured). However, to be fair, a previous assessment of the ESTRATEKO drop-net system including a bigger number of capture events resulted in 85% of successful capture events (i.e., wild boars did not appear in 15% of the planned capture events, hence the drop-net system could not be triggered) and when the wild boars made an appearance, multiple captures occurred in 94.1% of triggering events. These differences can be due to urban wild boar spatial ecology and seasonality^7,51^, as well as to suitable trap sites selection and monitoring during pre-baiting/capture periods^33^. Efficiency can be determined as the number of wild boars captured per successful capture event, or mean performance, which was higher in the aforementioned study (8.12 vs 5.3)^11^ and even higher during wild pig captures in the USA (10.7)^36^. Compared to cage or corral traps, and amongst live-capture methods aimed at wild boar, drop-nets have consistently been observed to display the highest performance^11,36^. Selectivity towards the targeted animals and operator safeness are also amongst the most common parameters used for capture method evaluation. Selectivity can be ensured through operator-mediated triggering or activation, which is the case of drop-nets, with the ability to prevent any stressful situation to any non-target species or individual. Other live-trapping methods may allow for the safe release of non-target captures, provided trapper professionalism, but in no case prevent the stressful episode supposed by restraint, which can result even in mortality of species or individuals particularly susceptible to capture myopathy^24,26^.

Finally, it deserves interest the adaptability of every capture method to different scenarios, which is a poorly evaluated but key aspect. This is probably due to the fact that, until recently, wildlife capture has been mostly performed in remote areas or landscapes with low levels of urbanization, which reduces public awareness and/or social concerns due to more utilitarian human values towards animals. In contrast, in more urbanized landscapes, human values tend to be more protectionist, where people are less supportive of killing animals and more supportive of protecting wildlife^14,52,53^. These circumstances can give room to social disturbances by animal sympathizers contrary to management measures based on wildlife capture and removal (population control). These disturbances may include opposing actions during captures performance, if found out, but more probably and difficult to prevent, deliberate vandalism of non-guarded traps, stealing of trap-related devices, such as camera-traps, or even freeing of the captured animals, which suppose a risk for the involved people. Cage and corral traps require longer deployment time of capture gear on field, which raises public awareness and increases the risk of vandalism when used in urban and peri-urban environments. This is the case for wild boar in the Metropolitan Area of Barcelona-MAB^39^, where most of these incidents have occurred when using cage and corral traps and ahead determined trap sites selection when using these methods (to avoid sites accessible to the general public) and limited capture success^11^. The operating protocol applied by ESTRATEKO S.L., including set up at dusk and dismantling at the end of the capture event, always during the night and before dawn, avoided any incident and resulted in increased and higher adaptability of the drop-net trap. This allowed the most suitable trap sites selection according to urban wild boar activity hotspots and maximized capture success. This adaptability may also apply to other non-urban scenarios that impose short times of trap system deployment.

## Conclusions

To summarize, (1) the application of the test procedures aimed at evaluating trapping standards suppose an opportunity to improve wildlife traps, capture protocols and welfare, being necessary their adaptation and that of acceptance thresholds to capture methods and species not considered previously; (2) to the best of our knowledge, this is the first assessment of welfare performance of a drop-net capture method according to international mammal trapping standards, resulting in the certification of the ESTRATEKO drop-net system for wild boar capture according to the AIHTS requirements. Although certification is not so clear when revisited according to newly proposed state-of-the-art standards, the need for adjusted test procedures and acceptance thresholds for restraining non-mechanical traps, namely for drop-nets, is clear. (3) Nonetheless, we suggest that a combined test procedure of both killing and restraining traps could be more suitable when using restraining traps by considering the parameter “time to unconsciousness” (either irreversible or not, if sacrifice is not the final objective of live-trapping) and establishing adapted thresholds. In any case, (4) to fully warrant animal welfare performance of drop-nets, the conditions to minimize time of handling or time to unconsciousness must be provided, preferably by ensuring a suitable number of skilled handlers. (5) Compared to other live-capture methods aimed at wild boar, drop-nets provoke a strong adrenergic acute stress response. However, the immediate assistance by handlers shortens the duration of the stressful situation. In addition, drop-nets maximize the probabilities of capturing entire sounders of prosocial species, which may be considered as more humane, as well as more efficient for population control purposes. (6) When properly used, drop-nets have the potential to easily coordinate minimum animal physical and mental distress with the highest values of capture success and maximum selectivity and adaptability to difficult environments and/or conditions. (7) Finally, other advantages of using drop-nets include easy technical management, operator safeness, low costs on maintenance over years and reduced trap visibility and distrust by targeted animals.

## Data Availability

Correspondence and requests for additional materials or information should be addressed to G.M. For additional information about ESTRATEKO, S.L.: https://www.ESTRATEKO.com.
